# Protection of Lycopene against Embryonic Anomalies and Yolk Sac Placental Vasculogenic Disorders Induced by Nicotine Exposure

**DOI:** 10.1155/2020/7957045

**Published:** 2020-06-06

**Authors:** Seul Gi Park, Chunmei Lin, Lee Wha Gwon, Jong-Geol Lee, In-Jeoung Baek, Beom Jun Lee, Sang-Yoon Nam

**Affiliations:** ^1^College of Veterinary Medicine and Veterinary Medicine Center, Chungbuk National University, Cheongju 28644, Republic of Korea; ^2^College of Chinese Medicine Materials, Jilin Agricultural University, Changchun, Jilin 130-118, China; ^3^Asan Institute for Life Sciences, University of Ulsan College of Medicine, Seoul 05505, Republic of Korea

## Abstract

Identification of a new agent from natural products for the protection of embryonic anomalies is potentially valuable. To investigate the protective effect exerted by lycopene against nicotine-induced malformations, mouse embryos in embryonic day 8.5 with yolk sac placentas were cocultured with 1 mM nicotine and/or lycopene (1 × 10^−6^, 1 × 10^−5^ *μ*M) for 48 h. The morphological defects and apoptotic cell deaths in the embryo and yolk sac placenta of the nicotine group were significantly increased. Exposure to nicotine resulted in reduced superoxide dismutase (SOD) activity and cytoplasmic SOD and cytoplasmic glutathione peroxidase mRNA levels, but increased lipid peroxidation level in embryos. Moreover, treatment with nicotine resulted in aggravated expressions of the mRNA or protein level of antiapoptotic (BCL2-associated X protein, B-cell lymphoma-extralarge, and caspase 3), anti-inflammatory (nuclear factor kappa-light-chain-enhancer of activated B cells and tumor necrosis factor-alpha), and vasculogenic (vascular endothelial growth factor-alpha, insulin-like growth factor-1, alpha smooth muscle actin, transforming growth factor-beta 1, and hypoxia inducible factor-1 alpha) factors in the embryo and yolk sac placenta. However, all the parameters were significantly improved by treatment with lycopene, as compared to the nicotine group. These findings indicate the potential of lycopene as a protective agent against embryonic anomalies and yolk sac vasculogenic and placenta-forming defects induced by nicotine through modulations of oxidative, apoptotic, vasculogenic, and inflammatory activities.

## 1. Introduction

Maternal smoking during early pregnancy has undesirable effects on the fetal organs and tissue development [1]. Moreover, the adverse effects of maternal smoking on embryonic development lead to low birth weight, premature labor, stillbirth, birth defects, and congenital malformations in the offspring [2]. Nicotine, a major toxic component among the thousands of substances present in cigarettes, is regarded as the causative agent of fetal anomalies [3]. Nicotine is quickly absorbed in the maternal blood, rapidly passes through the placenta, and builds up in the embryonic blood and amniotic fluid [4–6]. On reaching the fetus, nicotine induces ROS (reactive oxygen species), resulting in damage to the embryonic cells [7]. Thus, it is necessary to research the effects of smoking-induced malformation and identify natural preventive substances that can be consumed during pregnancy.

The yolk sac and placenta of rodents are structurally and functionally quite similar to those of humans [8–10]. Before the establishment of functional blood circulation in the hemotrichorial placenta, the embryo relies entirely on the yolk sac for nutrient supply [11]. The yolk sac is the first place where blood and blood vessels are formed, and its proper development is important for providing nutrients and supporting embryonic growth and development [12, 13]. Although the placenta secretes hormones and growth factors and protects the embryo from the maternal immune system, its primary function is the exchange of substances, such as oxygen, nutrients, and wastes, between mother and fetus [14, 15]. The mammalian developmental placenta largely consists of three layers: an outer layer containing uterine tissue, a middle junctional region where the fetal placenta meets the maternal blood vessels, and an inner layer containing numerous vascular branches [16]. A labyrinth, located in the middle layer of the mouse placenta, is the site where material exchange occurs between the embryo and mother [9, 17]. During mouse embryogenesis, the period of labyrinth development and primary villus formation is embryonic day (E) 8.5 to E 10.5 that is significant for the defection of embryonic disorders by toxicants [16]. Thus, it is essential to analyze morphological and biochemical changes in the embryo and placenta to understand the embryonic deformity caused by nicotine exposure during fetal organ formation.

Lycopene, an acyclic nonprovitamin A carotenoid, is plentiful in red fruits and vegetables, especially tomatoes, and is one of the most effective antioxidants among the dietary carotenoids because of its many conjugated double bonds [18–20]. Some studies report that lycopene is more efficacious than *β*-carotene and other carotenoids in removing singlet oxygen and ROS [21, 22]. Lycopene is also reported to prevent chronic diseases including cancers, cardiovascular disease, bone disorders, and neurodegenerative disease [23, 24]. However, although the numerous beneficial effects of lycopene are well-documented in disease control, its effectiveness against congenital abnormalities has not been demonstrated till date.

The present study, therefore, examines the adverse effects of maternal smoking on the embryo during embryonic organogenesis and investigates the suitability of lycopene as a natural substance in preventing nicotine-induced embryonic malformation.

## 2. Materials and Methods

### 2.1. Pregnant Animals

Eight-week-old ICR mice (Koatech) were used for mating. In the evening (20:00 hrs), male and female mice were placed in a cage, and the vaginal plug in the vulva was checked the following morning (08:00 hrs); this day was considered as embryonic day (E) 0.5 during further embryo culture. All experiments were performed as per the guidelines of the animal study guide (CBNUA, Guide for Care and Use of Animals).

### 2.2. Preparation for Culture Medium

Ten-week-old male Sprague–Dawley rats (Samtako, Osan, Korea) were used to prepare serum for culture medium [25]. Briefly, the collected blood samples were centrifuged twice for 10 min at 3600 × g. The purified serum supernatant was collected, after which the serum was inactivated at 56°C for 30 minutes and subsequently stored at –70°C. The serum was used after incubation at 37°C and filtering with a 0.2 *μ*m filter before use.

### 2.3. Embryo Culture and Treatment with Nicotine and Lycopene

The embryo culture system used in this experiment (Ikemoto Rika Kogyo, Tokyo, Japan) referred to a previously existing model [26]. Briefly, pregnant mice were sacrificed at E 8.5, and embryos with whole visceral yolk sacs and placentas were harvested. Prepared embryos were cultured for 48 h at 37 ± 5°C in medium (1 ml/1 embryo) supplemented with lycopene and/or nicotine. Based on our previous study, the concentration of nicotine was determined at 1 mM [27]. Lycopene (Sigma, St. Louis, MO, USA) used in the current experiment was dissolved in butylated hydroxyl toluene. Preliminary experiments were conducted by exposing the embryos to several concentrations of lycopene, in order to determine the effectual concentration of lycopene against nicotine-induced toxicity (data not shown). Consequently, two concentrations of lycopene (1 × 10^−6^, 1 × 10^−5^ *μ*M) were selected. Animals were randomly assigned to four experimental groups: control (Con), nicotine (N), nicotine and lycopene 1 × 10^−6^ *μ*M (N+Ly10^−6^), and nicotine and lycopene 1 × 10^−5^ *μ*M (N+Ly10^−5^). The culture bottles were gassed with a mixture of O_2_, CO_2_, and N_2_ (17 h: 5% O_2_, 5% CO_2_, and 90% N_2_; 7 h: 20% O_2_, 5% CO_2_, and 75% N_2_; and 24 h: 40% O_2_, 5% CO_2_, and 55% N_2_).

### 2.4. Embryonic Total Morphology Score Assessment

Embryos were evaluated immediately after the 48 h culture period, by applying the embryonic total morphology assessment [28]. The morphological evaluation was performed to score the growth and developmental changes of embryos and organ. The results were analyzed using the Kruskal–Wallis nonparametric ANOVA and Dunn's multiple comparison *post hoc* test.

### 2.5. Histopathological Evaluation

After the relevant culture period, samples were separated into embryos and yolk sac placentas and fixed in 4% paraformaldehyde. Paraplast-embedded tissues were sectioned at 5 *μ*m thickness and subsequently stained with hematoxylin and eosin (H&E).

Apoptotic cells were analyzed using the DeadEnd™ colorimetric TUNEL assay kit (Promega, WI, USA), according to our previous method [29]. Briefly, after deparaffinizing and rehydrating the slides, tissues were incubated in proteinase K (20 *μ*g/ml) for 10 min. Preequilibrated slides were labeled with TdT reaction mixture for 60 min at 37°C. The slides were washed in 2x SSC and blocked with 0.3% hydrogen peroxide for 5 min. After washing in 0.05% PBS-T (Tween 20), the slides were incubated with streptavidin/horseradish peroxidase-conjugated antibody (1 : 5000 in PBS) for 30 min. Apoptotic cells were visualized using DAB, and the nucleus of the cell was counterstained with hematoxylin.

### 2.6. Measurement of Lipid Peroxidation through MDA

Total protein was isolated and assessed from cultured embryos. Briefly, embryos were homogenized and the resultant supernatant was separated and mixed with 8.1% SDS, 20% acetic acid, and 0.75% 2-TBA; the mixture was heated for 30 min at 95°C. After cooling, the mixed solution was centrifuged, and the absorbance was measured at 532 nm and calculated from the 1,1,3,3,-tetramethoxypropane standard curve. The embryo protein content was calculated from the bovine serum albumin standard curve [30]. All experiments were evaluated according to our previous paper [25].

### 2.7. Superoxide Dismutase Activity Assay

Superoxide dismutase (SOD) activity in cultured embryos was measured using a SOD assay kit (Dojindo Laboratories, Kumamoto, Japan), according to our previously described method [25]. The results were expressed as the activity value divided by the amount of protein for one embryo.

### 2.8. Real-Time PCR Analysis

Treated embryos and placenta (including the yolk sac) were subjected to real-time PCR analysis, as per the protocol of our previous paper [25]. Briefly, total RNA was extracted using the Trizol kit (Invitrogen) and synthesized using cDNA Reverse Transcription Kit (AB, Applied Biosystems); the reaction was carried out using a 7500 Real-Time PCR System (Applied Biosystems). The primers used were vascular endothelial growth factor-alpha (VEGF-*α*), transforming growth factor-beta 1 (TGF-*β*1), hypoxia inducible factor-1 alpha (HIF-1*α*), insulin-like growth factor-1 (IGF-1), alpha smooth muscle actin (*α*-SMA), B-cell lymphoma-extralarge (Bcl-xL), tumor necrosis factor-alpha (TNF-*α*), SOD1, cytoplasmic glutathione peroxidase (GPx1), BCL2-associated X protein (Bax), caspase 3, and nuclear factor kappa-light-chain-enhancer of activated B cells (NF-*κ*B). In addition, glyceraldehyde-3-phosphate dehydrogenase (GAPDH) primers were used as an internal standard (Table 1). Data were calculated by nine independent assays using a comparative Ct method.

### 2.9. Western Blotting Analysis

Western blotting analysis was performed according to a previous paper [31]. Briefly, the cultured yolk sac placentas were homogenized in lysis buffer (PRO-PREP™ Protein Extraction Solution, iNtRON, Gyeonggi, Korea), centrifuged for 10 min at 4200 × g, and incubated at 95°C for 5 min in loading buffer. The prepared samples (120 *μ*g) were resolved on SDS-PAGE using 10% polyacrylamide gels and subsequently transferred to nitrocellulose membranes (Immobilon; Millipore, USA). Membranes were blocked for 1 h and immediately incubated with primary antibodies for VEGF-*α* (Abcam), TGF-*β*1 (Santa Cruz), and GAPDH (Santa Cruz). Probed membranes were subsequently incubated with the anti-rabbit secondary antibody (1 : 1000; Cell Signaling, MA, USA). Reactions were visualized using a Western Lightning Chemiluminescence reagent (PerkinElmer Life Science, MA, USA). Immunoreactive bands were quantified and normalized using an ImageJ software (Bethesda, MD, USA).

## 3. Results

### 3.1. Protective Effects of Lycopene in Morphological Defects

**Figure 1:**
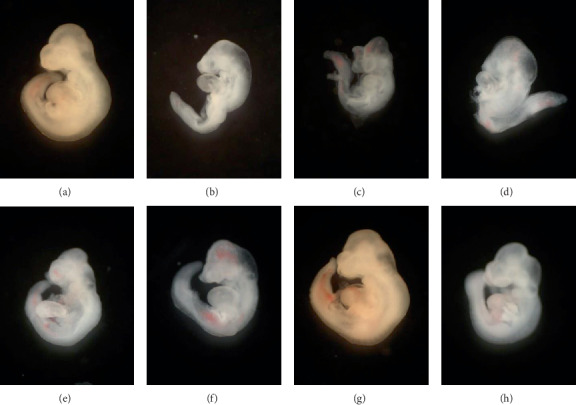
Morphological findings in embryos treated with nicotine or lycopene. (a) Normal control group. (b–d) Fetal malformations were observed in nicotine-treated embryos, such as open brain, reduced forebrain, abnormal rotation, and regressed forelimbs. (e–h) Coadministration of lycopene (1 × 10^−6^ and 1 × 10^−5^ *μ*M) results in embryo recovery, morphologically similar to the control group. Bar: 100 *μ*m.

Exposure to nicotine induces significant decreases in embryonic growth (Table 2) and developmental factors (Table 3 and Figure 1), as compared with the control group (*P* < 0.05). However, morphological scores altered by nicotine treatment were significantly inhibited in embryos cotreated with lycopene (Tables 2 and 3, Figure 1; *P* < 0.05).

**Figure 2:**
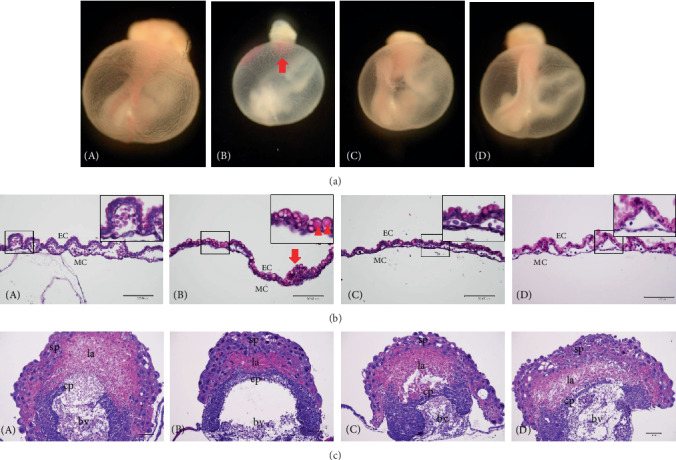
Effects of lycopene on defects of vascular branching in nicotine-induced yolk sac. (a) Yolk sac treated with nicotine shows defects such as growth retardation, bloodless color, no visible vascular network, and a ring of blood island at E 10.5. (b) Histological findings of the cultured yolk sac by hematoxylin and eosin staining. Yolk sac wall shows defects such as lack of vascular branching, blood island, and intracellular vacuoles. (c) Maternal and fetal border in the placenta. Lycopene attenuates nicotine-induced defects of the labyrinth in the placenta. A: control; B: nicotine; C, D: nicotine and lycopene (1 × 10^−6^ or 1 × 10^−5^ *μ*M). Sp: spongiotrophoblast; la: labyrinth; cp: chorionic plate; bv: blood vessel; EC: endothelial cell; MC: mesothelial cell; arrows: blood island; arrowheads: intracellular vacuole. Bar: 100 *μ*m.

Embryos treated with nicotine showed growth retardation, pale color, immature organization, and abnormal yolk sac morphology with marked reduction in size, impaired vascular branching, and a ring of blood islands around the chorioallantoic border in the placentas (Figure 2(a), B). In contrast, embryos cotreated with lycopene revealed a normal yolk sac structure with larger size, reddish color, complex vascular network, and disappearance of blood islands (Figure 2(a), C and D), similar to the control embryos (Figure 2(a), A).

In addition, the nicotine-exposed yolk sacs exhibited defects in histopathological as well as morphological evaluation. The nicotine group showed blood island, intracellular vacuoles, and abnormal blood vessel formation, such as absence of hematopoietic cells and disordered endodermal cells. In contrast, normal blood vessels filled with embryonic nucleated erythrocytes were observed in the lycopene cotreated groups. Likewise, nicotine-induced placenta showed deficiency of blood vessels where the allantois connects with the chorionic plate; moreover, the labyrinth, a layer filled with placental villi for the regulation of nutrient transport, was thin and poorly developed compared with control. In contrast, lycopene improved all defects of yolk sac placentas induced by nicotine treatment (Figures 2(a)–2(c), C and D).

### 3.2. Lycopene Protects Yolk Sac Placentas via Antiapoptotic and Antiproinflammatory Effects

**Figure 3:**
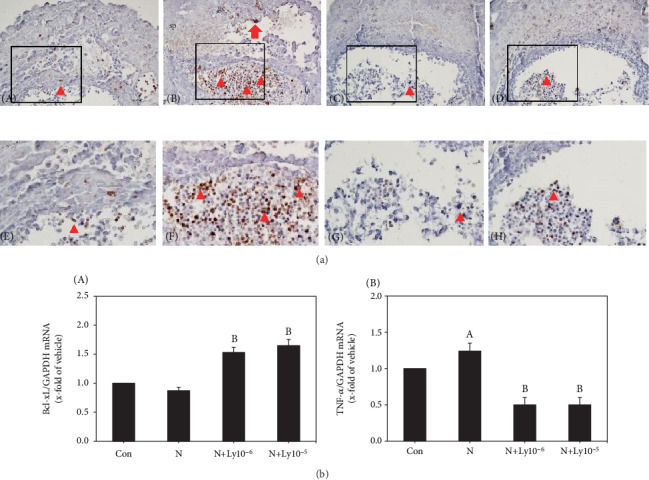
Lycopene protects the yolk sac placenta against nicotine-induced inflammation and apoptosis. (a) TUNEL-positive cells observed in nicotine-treated placentas. The enlarged structures of the boxed area. A, E: control group. B, F: nicotine group. C, G or D, H: nicotine and lycopene (1 × 10^−6^ or 1 × 10^−5^ *μ*M) groups, respectively. (b) A: Bcl-xL mRNA expression level. B: TNF-*α* mRNA expression level. Each value was normalized with GAPDH expression. Data represents average ± SEM. The lycopene groups were compared with the control (a) and nicotine (b) groups for significance (*P* < 0.05). Arrowheads: red blood cells; arrow: giant cells. Bar: 100 *μ*m.

TUNEL-positive cells in the yolk sac placenta treated with nicotine were fetal red blood cells and trophoblast giant cells (Figure 3(a), B and F). However, treatment with lycopene resulted in fewer positive fetal red blood cells, similar to the control group (Figure 3(a), C, D, G, and H).

The expression level of Bcl-xL mRNA in yolk sac placentas was 0.87 in the nicotine group. However, lycopene cotreated groups (1 × 10^−6^ or 1 × 10^−5^ *μ*M) revealed significantly increased values (1.53 or 1.65, respectively) (Figure 3(b), A).

In yolk sac placentas, the TNF-*α* mRNA levels were significantly increased to 1.24 in the nicotine group, which significantly decreased to 0.50 in response to cotreatment with lycopene (Figure 3(b), B).

### 3.3. Lycopene Regulates Expressions of Vasculogenic Factors in Nicotine-Induced Yolk Sac Placentas

**Figure 4:**
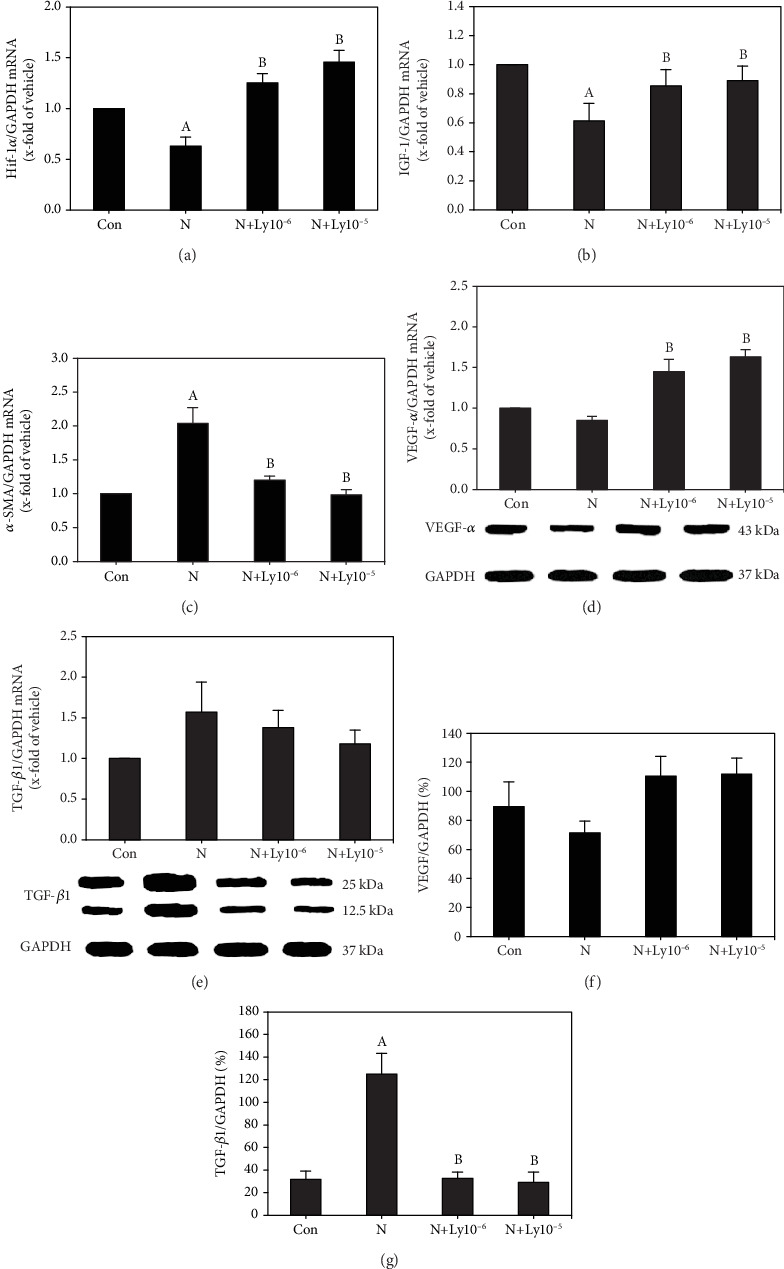
Lycopene regulates the expressions of growth factors in yolk sac placentas exposed to nicotine. (a) Hif-1*α* mRNA expression levels. (b) IGF-1 mRNA expression levels. (c) *α*-SMA mRNA expression levels. (d, e) mRNA expression levels of VEGF-*α* and TGF-*β*1, respectively. (f, g) Western blot analyses of VEGF-*α* and TGF-*β*1, respectively. The mRNA and protein values were normalized with GAPDH expression. Data represents average ± SEM. Lycopene cotreated groups were compared with control (a) and nicotine (b) groups for significance (*P* < 0.05).

Hif-1*α* mRNA expression of yolk sac placentas in the nicotine exposed group was significantly decreased to 0.63. However, cotreatment with lycopene (1 × 10^−6^ or 1 × 10^−5^ *μ*M) significantly recovered the values to 1.25 or 1.46, respectively (Figure 4(a)).

In yolk sac placentas, the IGF-1 mRNA level of the nicotine group (0.61) was significantly decreased as compared to that of the control group. In contrast, treatment with lycopene (1 × 10^−6^ or 1 × 10^−5^ *μ*M) significantly recovered the values to 0.85 or 0.89, respectively (Figure 4(b)).

The *α*-SMA mRNA level in yolk sac placentas of the nicotine group was significantly increased to 2.04, as compared to that of the control group. Cotreatment with lycopene (1 × 10^−6^ or 1 × 10^−5^ *μ*M) significantly decreases the *α*-SMA levels to 1.20 or 0.98, respectively (Figure 4(c)).

VEGF-*α* protein levels were decreased in yolk sac placentas exposed to nicotine, but cotreatment with lycopene resulted in significant increases. Likewise, the VEGF-*α* mRNA level in the yolk sac placenta was lower in the nicotine group (0.85) than in the control group; the mRNA levels significantly increased to 1.45 or 1.63 of levels obtained in the control group (Figures 4(d) and 4(f)) after treatment with lycopene (1 × 10^−6^ or 1 × 10^−5^ *μ*M, respectively).

TGF-*β*1 protein level was significantly increased in yolk sac placentas exposed to nicotine, but cotreatment with lycopene significantly decreased these levels. Also, the mRNA expressions in yolk sac placentas were increased in the nicotine group, but treatment with lycopene slightly recovered the damage caused by nicotine exposure (Figures 4(e) and 4(g)).

### 3.4. Lycopene Reduces Oxidative Stress and Increases Antioxidative Capacity in Nicotine-Induced Embryos

**Figure 5:**
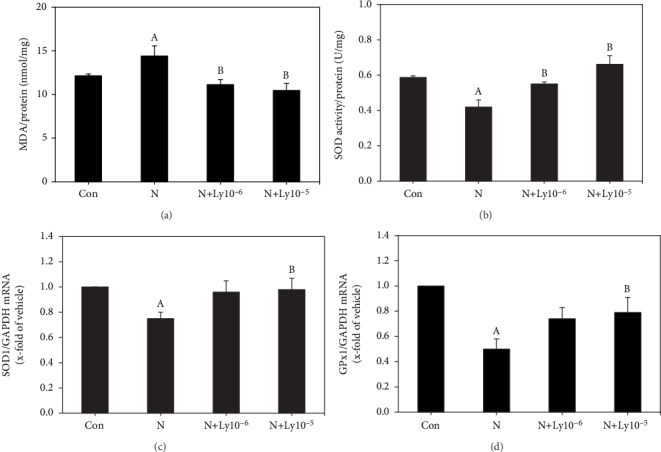
Lycopene reduces oxidative stress and increases antioxidant enzyme activity in nicotine-exposed embryos. (a) Lipid peroxidation was evaluated by malondialdehyde (MDA) concentration. (b) SOD activity levels. (c) SOD1 mRNA expression levels. (d) GPx1 mRNA expression levels. The mRNA results were normalized with GAPDH expression. Data are expressed as average ± SEM. Lycopene cotreated groups were compared with control (a) and nicotine (b) groups for significance (*P* < 0.05).

To investigate whether lycopene exerts an antioxidant effect on nicotine-induced teratogenesis, we analyzed lipid peroxidation, SOD activity, SOD1, and GPx1 mRNA expression patterns in embryos separated from the yolk sac placenta. Nicotine-exposed embryos exhibited significantly increased lipid peroxidation levels (14.4 ± 1.18 nmol/mg) when compared to the control group (12.1 ± 0.22 nmol/mg). However, treatment with lycopene (1 × 10^−6^ or 1 × 10^−5^ *μ*M) significantly reduced these levels to 11.1 ± 0.57 or 10.4 ± 0.81 nmol/mg, respectively (Figure 5(a)). Furthermore, mRNA levels of antioxidative markers and SOD activity in nicotine-induced embryos were significantly decreased to 0.75, 0.50, and 0.42 ± 0.04 U/mg, respectively. However, cotreatment with lycopene (1 × 10^−6^ or 1 × 10^−5^ *μ*M) and nicotine exposure resulted in increased SOD activity (0.55 ± 0.01 or 0.66 ± 0.05 U/mg), SOD1 (0.96 or 0.98), and GPx1 mRNA (0.74 or 0.79) levels, as compared to the nicotine treatment group (Figures 5(b)–5(d)).

### 3.5. Lycopene Controls the Expression of Apoptosis-Related Genes in Embryos Exposed to Nicotine

**Figure 6:**
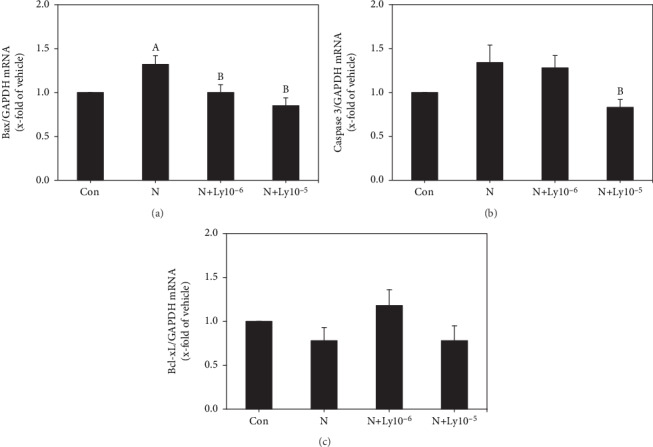
Gene expression changes of apoptosis-related genes (Bax, caspase 3, and Bcl-xL) in embryos using real-time PCR analysis. Each value was normalized with GAPDH expression. Data are expressed as average ± SEM. The mRNA levels of lycopene cotreated groups were compared with control (a) and nicotine (b) groups for significance (*P* < 0.05).

The Bax and caspase 3 mRNA levels in nicotine-exposed embryos were 1.32 and 1.34, respectively. Exposure to lycopene (1 × 10^−6^ or 1 × 10^−5^ *μ*M) resulted in decreased mRNA levels of Bax (1.00 or 0.85, respectively) and caspase 3 (1.28 or 0.83, respectively) (Figures 6(a) and 6(b)). Similarly, Bcl-xL mRNA levels in the nicotine group decreased to 0.78 as compared to the control, but increased to 1.18 when exposed to lycopene (Figure 6(c)).

### 3.6. Lycopene Adjusts Gene Expression of Proinflammatory Cytokines in Nicotine-Exposed Embryos

**Figure 7:**
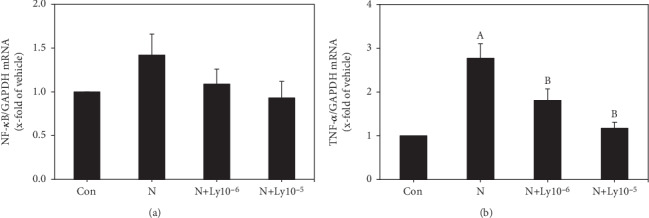
Expression changes of NF-*κ*B and TNF-*α* mRNA levels in embryos exposed to nicotine (N; 1 mM) and/or lycopene (Ly; 1 × 10^−6^ and 1 × 10^−5^ *μ*M). Each value was normalized with GAPDH expression. Data are expressed as average ± SEM. Lycopene cotreated groups were compared with control (a) and nicotine (b) groups for significance (*P* < 0.05).

Exposure of embryos to nicotine induces an increase in the NF-*κ*B mRNA level (1.42), as well as significant increase in the TNF-*α* mRNA level (2.77) when compared to the control group. However, the addition of lycopene (1 × 10^−6^ or 1 × 10^−5^ *μ*M) decreases the NF-*κ*B (1.09 or 0.93, respectively) and TNF-*α* (1.81 or 1.17, respectively) mRNA levels (Figures 7(a) and 7(b)).

## 4. Discussion

The first trimester in pregnancy is considered an important period during development, since the embryo is sensitive to environmental influences and is active for organogenesis [4, 32]. Therefore, we cultured E 8.5 embryos to embryonic day 10.5, which is the active stage for organogenesis in mice. We found that exposure of early embryos to nicotine during this period resulted in decreased embryo growth and development of all organs, as compared to controls. However, supplementation of lycopene (1 × 10^−6^ or 1 × 10^−5^ *μ*M) with nicotine significantly increased most of the growth and development scores. These results indicate that lycopene is effective in protecting against prenatal morphological defects due to nicotine exposure.

Yolk sac placenta developments are important during organogenesis [12, 13]. Yolk sac comprises an outer layer of visceral endoderm, the inner mesothelial cell (MC) and endothelial cell (EC) layers, and blood vessels [33–35]. Before the establishment of functional blood circulation in the placenta, the embryo relies entirely on the yolk sac for nutrient supply [11]. Accordingly, in order to support embryonic growth and development, the appropriate development of yolk sac is a priority. Likewise, normal development of the placenta is essential for embryonic development, especially the labyrinth, which is the site of efficient nutrient exchange between mother and fetus [36]. Histological examination and biochemical observation of yolk sac placentas are therefore necessary to help us understand malformations induced by foreign materials. In the current study, yolk sacs treated with nicotine alone were of smaller size, pale color, and immature blood vessel formation, and the yolk sac wall showed paucity of vessels, blood island, and intracellular vacuoles. In addition, histological examination of placentas treated with nicotine showed defects in labyrinth formation. In contrast, cotreatment with lycopene improved the vascular network and labyrinth formation. Moreover, TUNEL assay revealed that apoptotic cells were scattered in the fetal RBCs of the labyrinth and giant cells in the nicotine-exposed group, but supplementation with lycopene remarkably decreased the TUNEL-positive cells, as well as showed significant recovery in the mRNA levels of TNF-*α* and Bcl-xL. These findings indicate that lycopene improves the defects of yolk sac vascularization, formation of the labyrinth, and fetal hematopoiesis induced by nicotine exposure.

During early organogenesis, the uterine environment is relatively hypoxic [37]. Hypoxia induces oxidative stress, and the early embryo is sensitive to this oxidative stress environment [38, 39]. Hif-1 is activated during hypoxia and modulates the transcription of genes that play important functions in energy metabolism, erythrogenesis, vasculogenesis, and cell survival. As a result, it regulates the vascular development-related gene, such as vascular endothelial growth factor (VEGF) [40]. Moreover, the lack of Hif activity results in the loss of syncytiotrophoblasts in the mouse placenta [41]. Therefore, downregulation of Hif-1*α* in the nicotine-exposed embryos results in abnormal organogenesis. Furthermore, the nicotine-induced abnormal Hif-1*α* transcription in yolk sac placenta subsequently upregulates TGF-*β*1 and downregulates VEGF-*α* and IGF-1. These abnormal gene expressions result in defects of vasculogenesis and formation of the labyrinth. However, cotreatment with lycopene significantly improved these defects.

Some studies revealed that nicotine increases the ROS expression, causing oxidative damage and growth retardation in embryos [7]. Oxidative stress leads to embryonic malformation attributed to cell membrane, protein, and DNA damages, along with apoptosis [42]. In this study, the addition of lycopene to nicotine-exposed embryos greatly recovered the mRNA expression levels of apoptotic markers (Bax, caspase 3, and Bcl-xL) and anti-inflammatory cytokines (TNF-*α* and NF-*κ*B). Furthermore, since oxidative stress induces apoptosis and inflammation during early organogenesis caused by nicotine, it is important to protect the embryos from exposure to oxidative stress during early embryogenesis. In the current study, lycopene cotreatment with nicotine improved lipid peroxidation, SOD activity, and antioxidant enzyme (SOD1, GPx1) mRNA expressions. These findings indicate that lycopene reduces the nicotine-induced oxidative stress, but increases antioxidative activities in embryos.

## 5. Conclusions

In conclusion, the findings from our study indicate that lycopene plays an antioxidative, vasculogenic, antiapoptotic, and anti-inflammatory roles in both embryos and yolk sac placentas damaged by nicotine exposure during early embryogenesis, which is an important stage of development. Hence, lycopene has the potential to be effectively used in the pharmaceutical market as a natural preventive agent that is efficacious in protecting embryos from smoking-induced risks during pregnancy.

## Figures and Tables

**Table 1:** Primer sequences used in this study.

Primer	Sequence
VEGF-*α*	
Forward	5′-AACGATGAAGCCCTGGAGTG-3′
Reverse	5′-GCTGGCTTTGGTGAGGTTTG-3′
TGF-*β*1	
Forward	5′-TGGAGCAACATGTGGAACTC-3′
Reverse	5′-CAGCAGCCGGTTACCAAG-3′
HIF-1*α*	
Forward	5′-CACCAGACAGAGCAGGAA-3′
Reverse	5′-TCAGGAACAGTATTTCTTTGATTCA-3′
IGF-1	
Forward	5′-TCGGCCTCATAGTACCCACT-3′
Reverse	5′-ACGACATGATGTGTATCTTTATTGC-3′
*α*-SMA	
Forward	5′-TCCAGCCATCTTTCATTGGG-3′
Reverse	5′-TGGTACCCCCTGACAGGAC-3′
Bcl-xL	
Forward	5′-TGACCACCTAGAGCCTTGGA-3′
Reverse	5′-TGTTCCCGTAGAGATCCACAA-3′
TNF-*α*	
Forward	5′-TACCTTGTTGCCTCCTCTT-3′
Reverse	5′-GTCACCAAATCAGCGTTATTAAG-3′
SOD1	
Forward	5′-TGCGTGCTGAAGGGCGAC-3′
Reverse	5′-GTCCTGACAACACAACCTGGTTC-3′
GPx1	
Forward	5′-TGTTTGAGAAGTGCGAAGTG-3′
Reverse	5′-GTGTTGGCAAGGCATTCC-3′
Bax	
Forward	5′-CTCAAGGCCCTGTGCACTAA-3′
Reverse	5′-CACGGAGGAAGTCCAGTGTC-3′
Caspase 3	
Forward	5′-AAAGCCGAAACTCTTCATCAT-3′
Reverse	5′-GTCCCACTGTCTGTCTCA-3′
NF-*κ*B	
Forward	5′-CACTGCTCAGGTCCACTGTC-3′
Reverse	5′-CTGTCACTATCCCGGAGTTCA-3′
GAPDH	
Forward	5′-CGTGCCGCCTGGAGAAACC-3′
Reverse	5′-TGGAAGAGTGGGAGTTGCTGTTG-3′

**Table 2:** Growth changes in the cultured mouse embryos.

Group	Con	N	N+Ly10^−6^	N+Ly10^−5^
No. of embryos	26	30	33	25
Yolk sac diameter (mm)	3.35 ± 0.10	2.86 ± 0.06^a^	3.25 ± 0.08^b^	3.39 ± 0.11^b^
Crown-rump length (mm)	2.83 ± 0.10	2.36 ± 0.05^a^	2.60 ± 0.05^b^	2.76 ± 0.07^b^
Head length (mm)	1.51 ± 0.14	1.27 ± 0.09^a^	1.18 ± 0.02	1.31 ± 0.04^b^
No. of somites	31.69 ± 0.33	29.27 ± 0.28^a^	31.09 ± 0.24^b^	31.12 ± 0.37^b^

Each value represents the mean ± SEM. ^a^vs. normal control (Con) group at *P* < 0.05. ^b^vs. nicotine (N) group at *P* < 0.05. Ly: lycopene.

**Table 3:** Developmental changes in the cultured mouse embryos.

Group	Con	N	N+Ly10^−6^	N+Ly10^−5^
No. of embryos	26	30	33	25
Yolk sac circulatory	4.15 ± 0.06	3.78 ± 0.03^a^	4.08 ± 0.03^b^	4.10 ± 0.05^b^
Allantois	2.18 ± 0.06	1.75 ± 0.04^a^	2.08 ± 0.03^b^	2.15 ± 0.06^b^
Flexion	4.92 ± 0.06	4.34 ± 0.20	4.98 ± 0.02	4.89 ± 0.08
Heart	4.86 ± 0.07	3.85 ± 0.13^a^	4.68 ± 0.08^b^	4.71 ± 0.06^b^
Hindbrain	4.40 ± 0.08	3.73 ± 0.14^a^	4.24 ± 0.04^b^	4.28 ± 0.06^b^
Midbrain	4.67 ± 0.09	3.82 ± 0.14^a^	4.31 ± 0.04^b^	4.42 ± 0.06^b^
Forebrain	5.50 ± 0.15	4.03 ± 0.17^a^	4.71 ± 0.09^b^	4.83 ± 0.06^b^
Otic system	4.91 ± 0.08	3.89 ± 0.17^a^	4.61 ± 0.07^b^	4.79 ± 0.06^b^
Optic system	4.86 ± 0.07	3.83 ± 0.15^a^	4.52 ± 0.06^b^	4.56 ± 0.06^b^
Branchial bars	3.73 ± 0.06	2.88 ± 0.07^a^	3.32 ± 0.08^b^	3.44 ± 0.06^b^
Maxillary process	2.83 ± 0.07	1.91 ± 0.11^a^	2.40 ± 0.07^b^	2.36 ± 0.06^b^
Mandibular process	2.71 ± 0.10	1.83 ± 0.09^a^	2.29 ± 0.07^b^	2.29 ± 0.06^b^
Olfactory system	2.70 ± 0.13	1.20 ± 0.15^a^	1.87 ± 0.14	1.96 ± 0.06^b^
Caudal neural tube	5.00 ± 0.00	4.80 ± 0.07	5.00 ± 0.00	5.00 ± 0.06^b^
Forelimb	2.70 ± 0.07	2.14 ± 0.10^a^	2.59 ± 0.08^b^	2.70 ± 0.06^b^
Hind limb	1.04 ± 0.13	0.16 ± 0.05^a^	0.53 ± 0.09^b^	0.54 ± 0.06^b^
Somites	4.12 ± 0.06	3.87 ± 0.06	4.00 ± 0.00	4.04 ± 0.06^b^
Total score	72.98 ± 1.21	58.30 ± 1.65	67.26 ± 0.78	68.52 ± 1.10

Each value represents the mean ± SEM. ^a^vs. normal control (Con) group at *P* < 0.05. ^b^vs. nicotine (N) group at *P* < 0.05. Ly: lycopene.

## Data Availability

The data used to support the findings of this study are included within the article.
